# Arthroscopic Glenoid Bone Tunnel and Half Bundle of Peroneus Longus Tendon‐Based Subscapular Sling Procedure and Labral Reconstruction for Anterior Shoulder Instability

**DOI:** 10.1002/atn2.70246

**Published:** 2026-08-27

**Authors:** Yue Geng, Jiangtao Gao, Xusheng Bo, Mingsi Yu, Yi Zheng, Zhuangdai Zhang, Jiangtao Dong

**Affiliations:** ^1^ The Third Hospital of Hebei Medical University Shijiazhuang Hebei China

## Abstract

The arthroscopic subacromial sling procedure is used to treat recurrent anterior shoulder instability. As an arthroscopic technique with less potential for complications, the subscapular sling with a half bundle of the peroneus longus tendon provides both dynamic and static stability. This procedure uses the half bundle of the peroneus longus tendon graft as a sling around the upper two‐thirds of the subscapular tendon, with the graft attached to the anterior glenoid rim through the glenoid bone tunnel. We change the method of obtaining and fixing the graft during the subscapular sling procedure. The half bundle of the peroneus longus tendon has sufficient strength and diameter and reduces the risk of complications. The establishment of the glenoid bone tunnel can provide a more stable fixation for the graft, facilitating stable tendon‐bone healing. This arthroscopic subscapular sling procedure with glenoid bone tunnel and half bundle of the peroneus longus tendon graft can be considered an alternative to existing surgical treatment options for recurrent anterior shoulder instability.

## Graphical abstract

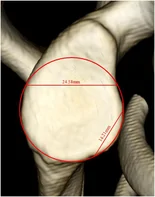

**VIDEO 1:** Arthroscopic glenoid bone tunnel and half bundle of peroneus longus tendon‐based subscapular sling procedure for anterior shoulder instability. First, establish the standard anterior portal, posterior portal, anterior‐lateral portal, and anterior‐superior portal. A diagnostic arthroscopic examination is performed through the posterior portal, especially the subscapular tendon. The key step is to thoroughly clear the soft tissues at the 2 and 5 o'clock positions of the anterior glenoid. Make a 2 to 3 cm incision on the posterior lateral ankle. Expose the peroneus longus tendon (PLT) and divide it into 2 parts. Measure the length and diameter of the PLT. One end of the tendon is woven, whereas the other end is left unsewn to adjust the PLT length. A switching stick is used to locate the incision site of the subscapular tendon. Turn the arthroscope to the subacromial view, expose the subscapular tendon and switching stick, and make a slit. The guide suture passes through the subscapular tendon. Place the tunnel‐locating device at the 2 o'clock position on the anterior glenoid. Create the glenoid bone tunnel and place 2 guide sutures. Place the suture anchors at the 3 and 5 o'clock positions on the anterior glenoid to reconstruct the anterior glenoid labrum. Using the guide sutures, the graft is pulled through the subscapular tendon and into the glenoid tunnel. After confirming the stability and containment of the joint, fix the graft sutures at the entrance of the tunnel using the miniplate. Finally, it is observed that the transferred PLT could effectively reconstruct the anterior labrum and the subscapular sling, restoring the stability and containment of the shoulder. Video content can be viewed at https://doi.org/10.1002/atn2.70246.

For anterior shoulder instability, the treatment depends on the severity of the damage to the anterior structures.^1^, ^2^ All common surgical techniques (Bankart and bone‐grafting procedures) have their own distinct complications.^3^, ^4^, ^5^ In pursuit of new treatment options, the arthroscopic subscapular sling procedure has been developed. Through biomechanical and clinical studies, the subscapular sling procedure has shown a favorable stability effect.^6^, ^7^, ^8^ However, the length, strength, and reliable fixation of the graft remain key points that need to be explored. Therefore, the purpose of this article is to present a half bundle of the peroneus longus tendon (PLT) graft based on the glenoid bone tunnel, to construct the subscapular sling and reconstruct the labrum, and to ensure appropriate subscapular sling tension and reliable tendon‐bone healing of the graft, providing static and dynamic stabilization of the shoulder. Indications and contraindications are listed in Table 1.

**TABLE 1:** Indications and Contraindications

Indications	Contraindications
Mere anterior soft‐tissue damage	Posterior and multidirectional instability
Minimal glenoid bone loss	Severe glenoid bone loss
Residual anterior shoulder instability	Torn subscapular tendon
Intact subscapular tendon	Peroneus longus tendon tear

## SURGICAL TECHNIQUE

This article focuses on arthroscopic glenoid bone tunnel and half bundle of PLT‐based subscapular sling procedure and labral reconstruction. Our surgical technique is presented in Video 1.

### Anesthesia and Patient Setup

**FIGURE 1:**
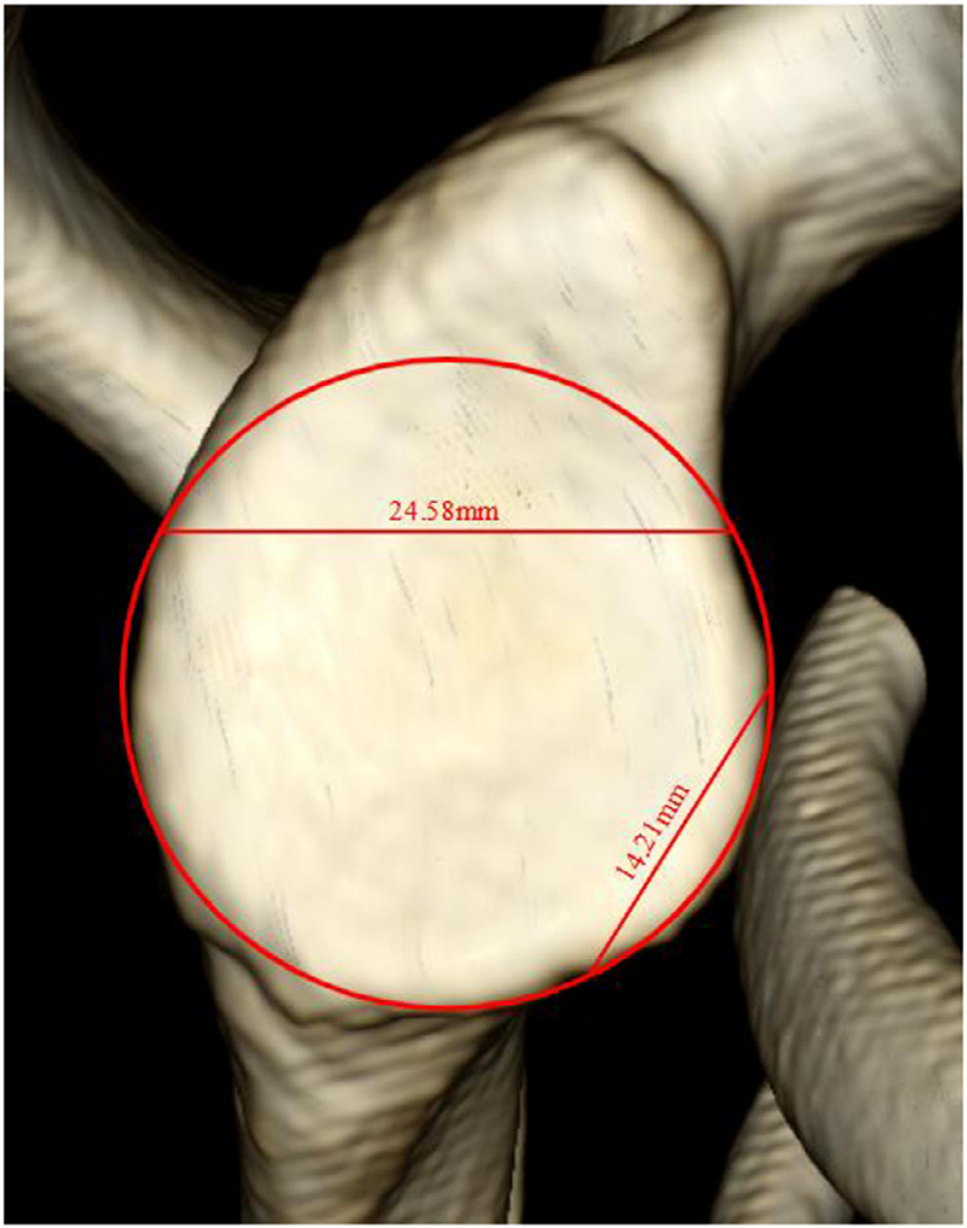
Preoperative preparation: predict the length of the graft. The length of the glenoid bone tunnel: confirm the entrance (at the 10 o'clock position on the posterior glenoid) and exit (at the 2 o'clock position on the anterior glenoid) of the bone tunnel, and measure the shortest distance between the 2 positions. Length of the glenoid bone tunnel: 24.58 mm. The length of the graft required for labral reconstruction: confirm the position of the anterior labral reconstruction (at the 3 o'clock position to the 5 o'clock position on the anterior glenoid) and measure the shortest distance between the 2 positions. The length of the graft required for labral reconstruction is approximately 14.21 mm. Length of the graft: 70 mm. Length of the woven graft: 20 mm.

Preoperative preparation: The glenoid width is measured by computed tomography with three‐dimensional reconstruction and the graft length is estimated. The length of the graft needs to be sufficient for the labrum reconstruction and tendon‐bone healing based on the bone tunnel. The length of the glenoid bone tunnel is approximately 25 mm. The length of the graft required for labral reconstruction is approximately 15 mm (Figure 1). The length of the graft within the tunnel is at least 15 mm. The tunnel is located 5 mm below the glenoid surface. Therefore, the total length of the graft should be 70 mm.

**FIGURE 2:**
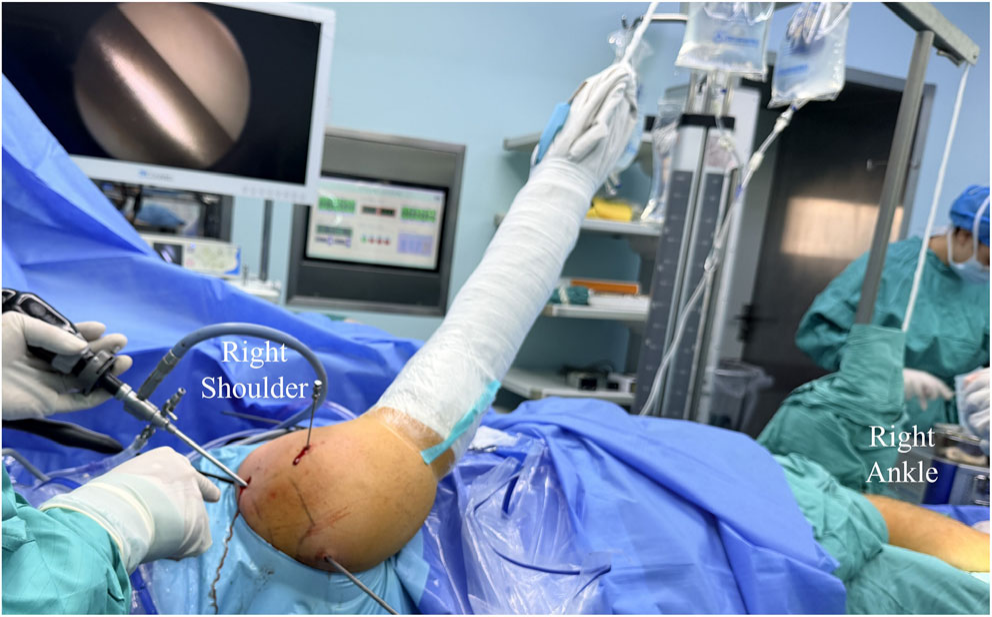
Patient positioning: right shoulder in the lateral decubitus position. Expose the right shoulder and the right ankle.

The surgical procedure is performed with the patient under general and locoregional anesthesia (interscalene block and adrenaline local anesthesia). The patient is placed in the lateral decubitus position (Figure 2) as it enables the shoulder to maintain an internal rotation of at least 20° to avoid impingement of the graft between the humeral head and the subscapular tendon, and the graft is easily harvested from the ipsilateral ankle. The advantage of the lateral decubitus position can reduce the risk of hypotension, bradycardia.

#### Step 1: Diagnostic Arthroscopy and Initial Processing

**FIGURE 3:**
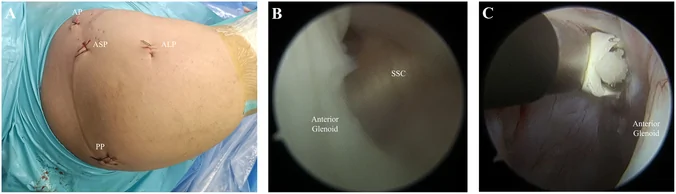
Creation of operational portal and diagnostic arthroscopy. (A) The portals used for the arthroscopic approach are created: AP, PP, ALP, and ASP. (B) Intracapsular diagnostic arthroscopy from the posterior viewing portal in a right shoulder in the lateral decubitus position using a 30° arthroscope, especially the SSC and anterior glenoid labrum. (C) Intracapsular view from the anterior‐superior viewing portal in a right shoulder in the lateral decubitus position, with the AP as the working portal. Thoroughly clear the soft tissues at the 2 and 5 o'clock positions of the anterior glenoid. (ALP, anterior‐lateral portal; AP, anterior portal; ASP, anterior‐superior portal; PP, posterior portal; SSC, subscapular tendon.)

Based on the anatomical landmarks of the shoulder, establish the standard anterior portal (AP), posterior portal (PP), anterior‐lateral portal, and anterior‐superior portal (ASP) (Figure 3A). A diagnostic arthroscopic examination is performed, and the rotator cuff and in particular the subscapular tendon are inspected to rule out injuries (Figure 3B). Furthermore, the amount of glenoid bone loss, on/off‐track Hill‐Sachs lesions, and ligament tears are assessed.

A shaver and a radiofrequency device are used to thoroughly remove the inflammation around the subscapular tendon and identify the axillary nerve to keep it at a distance from the portals and instruments during surgery. The key step is to thoroughly clear the soft tissues and remaining labrum at the 2 and 5 o'clock positions of the anterior glenoid to facilitate the positioning of the bone tunnel and firm attachment of the graft (Figure 3C). Instrumentation is performed lateral to the conjoined tendon, thus preserving the conjoined tendon and musculocutaneous nerve.

#### Step 2: Preparation of the PLT

**FIGURE 4:**
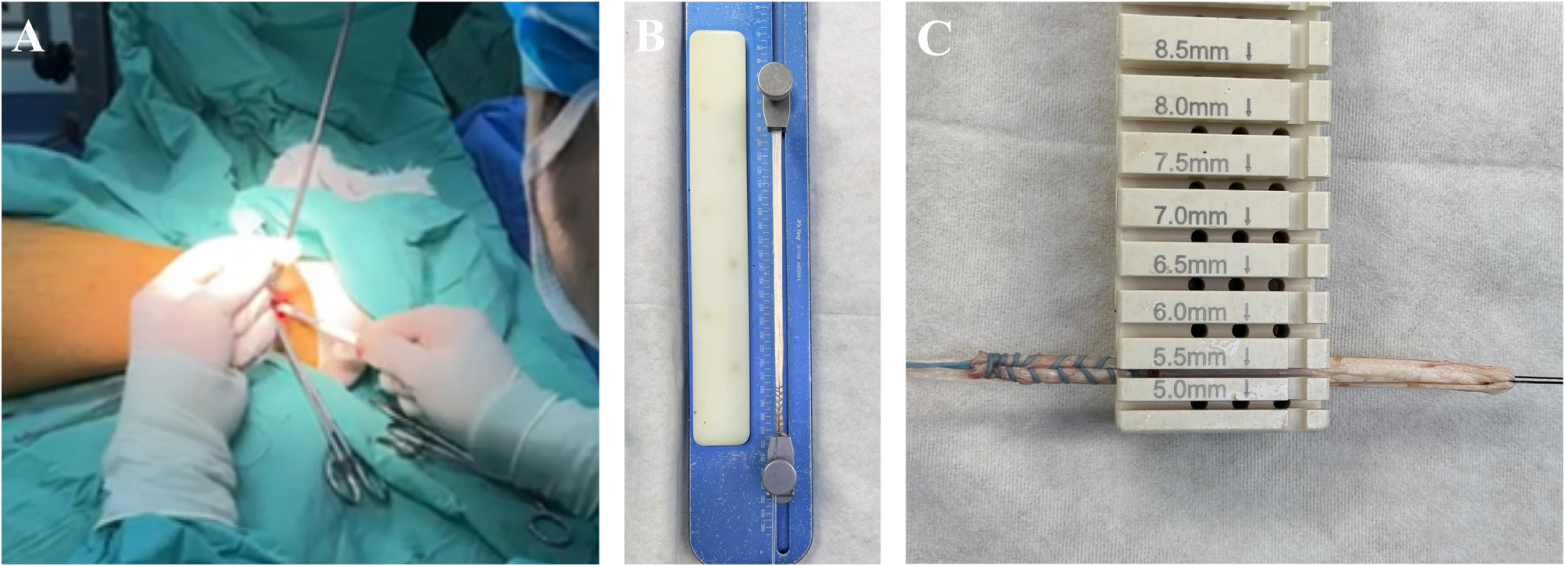
Preparation of the PLT. (A) Harvesting of the PLT through the incision on the posterior lateral ankle styloid in the right lateral decubitus position. (B) Measure the length of the PLT. The total length of the PLT is 240 mm, and the required sufficient length is approximately 70 mm, with the weaving length being 20 mm. (C) Measure the diameter of the PLT. The diameter of the PLT is 5.5 mm. (PLT, peroneus longus tendon.)

A 2 to 3 cm skin incision is made on the posterior lateral ankle styloid process. Separate the fascia and expose the PLT. Under visual observation, use a knife to evenly divide the PLT into 2 parts and cut 1 part as close to the distal side as possible (Figure 4A). Use a tendon stripper to extract the tendon toward the proximal end of the PLT. Measure the length and diameter of the PLT and perform the trimming (Figure 4B,C). To ensure that the graft has sufficient length, a high‐strength suture is used to weave at 1 end of the PLT, whereas the other end is left unsewn to allow for adjustment of the PLT length.

#### Step 3: Arthroscopic Transport and Fixation of the Graft

**FIGURE 5:**
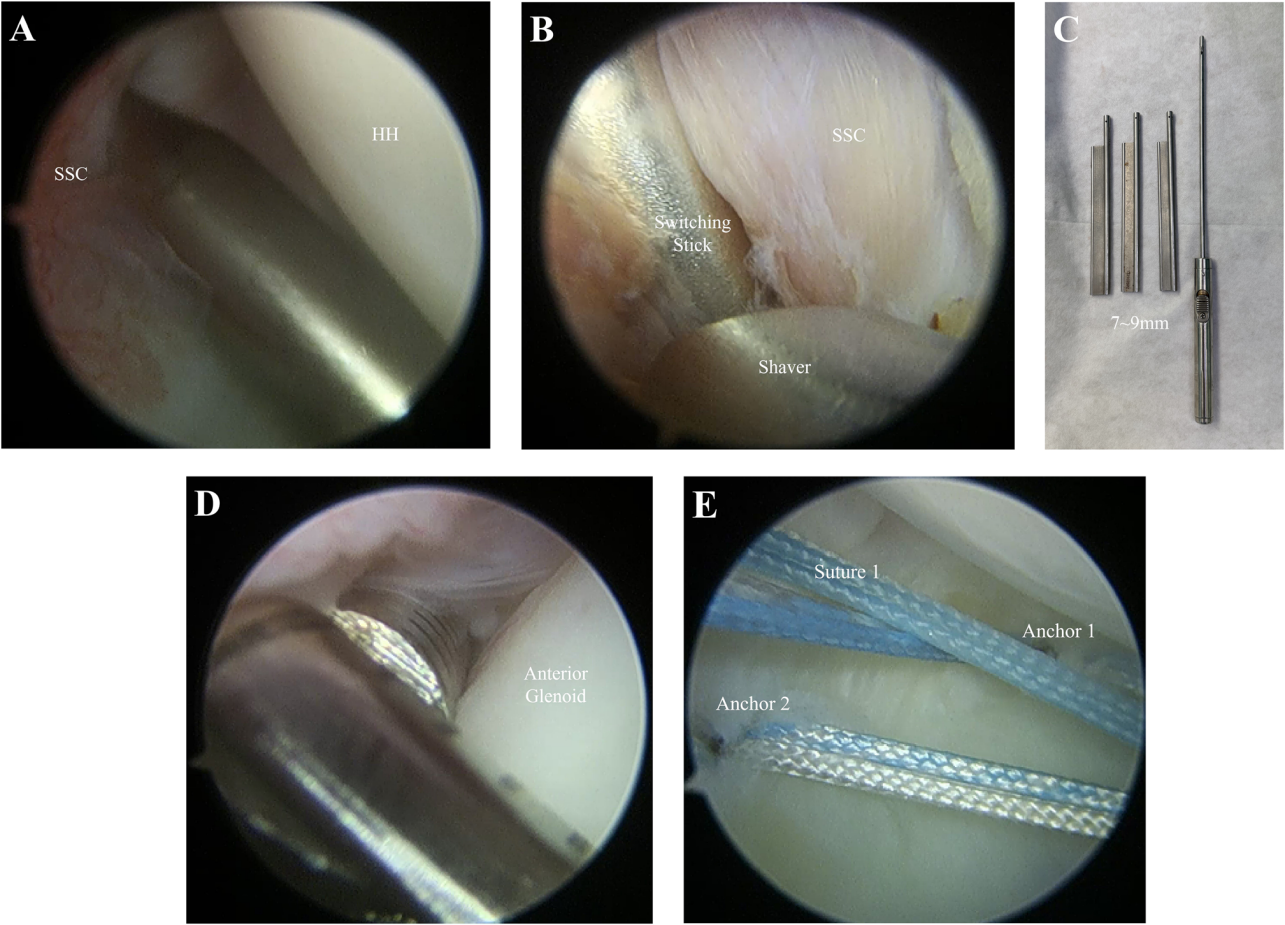
Preparation before graft transfer. (A) Intracapsular view from the anterior‐superior viewing portal in a right shoulder in the lateral decubitus position, with the posterior portal as the working portal. Use a switching stick to locate the incision site of the SSC. (B) Subacromial view from the anterior‐lateral viewing portal in a right shoulder in the lateral decubitus position, with the anterior‐superior portal as the working portal. Make a slit in the longitudinal direction between the lower and middle third of the subscapular tendon. (C) Tunnel‐locating device needed to perform the glenoid bone tunnel procedure. Select the appropriate tunnel‐locating device based on the diameter of the graft. (D) Intracapsular view from the anterior‐superior viewing portal in a right shoulder in the lateral decubitus position, with the posterior portal as the working portal. Place the tunnel‐locating device at the 2 o'clock position on the anterior glenoid. (E) Intracapsular view from the anterior‐superior viewing portal in a right shoulder in the lateral decubitus position, with the anterior portal as the working portal. Place the anchors at the 3 and 5 o'clock positions of the glenoid and tidy up the sutures, as shown in the figure. (HH, humeral head; SSC, subscapular tendon.)

Under the observation view of ASP, a switching stick through the PP is used to locate the incision site of the subscapular tendon (Figure 5A). Keep the switching stick in its position and turn the arthroscope to the subacromial view through the anterior‐lateral portal. Use the radiofrequency device to expose the subscapular tendon and the switching stick, and make a slit in the longitudinal direction between the lower and middle third of the subscapular tendon (Figure 5B). Replace the switching stick with the instrument portal, with the help of a grasper; guide suture 1 is introduced into the joint from the PP through the slit in the subscapular tendon and back out through the ASP. Placing the guiding suture 1 facilitates the placement of the graft. Select a tunnel‐locating device of the appropriate diameter based on the graft to prevent the rupture of the bone tunnel (Figure 5C). From the PP, place the appropriate tunnel‐locating device at the 2 o'clock position on the anterior glenoid (Figure 5D). Confirm the exit of the glenoid bone tunnel. Sequentially use the tunnel aiming pin and drill to create the glenoid bone tunnel, after which guide sutures 2 and 3 are introduced into the joint from the PP through the glenoid bone tunnel and back out through the AP. From the AP, place 2 suture anchors (Rejon, Zhejiang, China) at the 3 and 5 o'clock positions on the glenoid. To reduce the interference from sutures, half of the sutures on the anchors back out through the PP (Figure 5E), and guide suture 1 in the PP is passed between the 2 parts of the sutures on the anchors and back out through the AP, allowing the surgeon (D.J.T.) to determine the number and position of the guide sutures.

**FIGURE 6:**
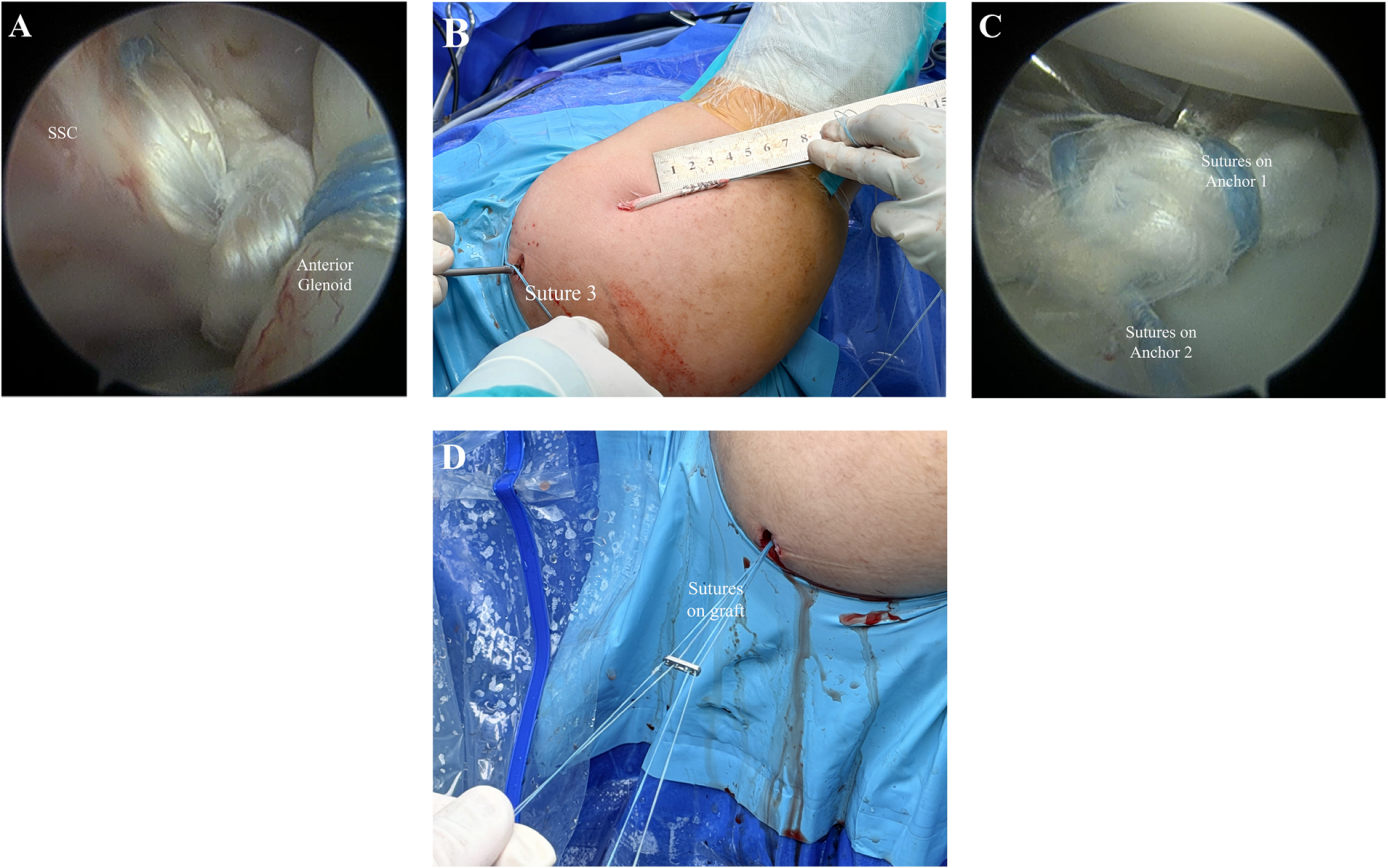
Transplantation and fixation of the grafts. (A) Intracapsular view from the anterior‐superior viewing portal in a right shoulder in the lateral decubitus position, with the posterior portal as the working portal. Graft exits from the posterior approach through the bone tunnel. The graft passes through the SSC and enters the glenoid bone tunnel. (B) Weave the other end of the graft. (C) Intracapsular view from the anterior‐superior viewing portal in a right shoulder in the lateral decubitus position, with the anterior portal as the working portal. Fix the medial graft of the subscapular tendon using suture anchors. (D) Use a miniplate to firmly fix the graft sutures. (SSC, subscapular tendon.)

**FIGURE 7:**
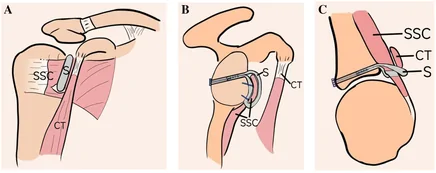
Schematic drawings of the subscapular sling. (A) Coronal image. (B) Sagittal image. (C) axial image. (CT, conjoined tendon; S, sling; SSC, subscapular tendon.)

The end suture of the woven graft is connected to guide suture 1 in the AP and pulled into the joint between the 2 parts of the sutures on the anchors through the slit in the subscapular tendon and back out through the ASP. The end suture of the woven graft is connected to guide suture 2 in the anterior‐superior pulled into the bone tunnel and back out through the PP (Figure 6A). The woven graft is experimentally pulled into the tunnel. To leave the appropriate length within the bone tunnel, the overall length and tension of the graft can be adjusted according to the length of the graft that has not been pulled into the joint. After determining the length and tension of the graft, the other end of the graft is woven (Figure 6B). The arthroscope is moved to the subacromial view. With the help of a grasper, the other end of the graft is introduced into the joint between the anterior wall of the subscapular tendon and the coracoid from the AP and back out through the ASP. The other end suture of the graft is connected to the guide suture 3 in the anterior‐superior pulled into the bone tunnel and back out through the PP. Both ends of the graft are pulled into the bone tunnel. After adjusting the position of the graft, the graft between the 2 parts of the sutures on the anchors is reliably fixed to the anterior glenoid rim through knotting to reconstruct the anterior glenoid labrum (Figure 6C). Tighten the end of the graft, and after confirming the stability and containment of the joint, fix the graft sutures at the entrance of the bone tunnel using the miniplate (Arthrex, Naples, FL) (Figure 6D). Figure 7 presents schematic drawings of the subscapular sling.

### Postoperative Care and Rehabilitation

The patient uses a supportive sling for 4 weeks. Active exercises begin 6 weeks after the operation. Magnetic resonance imaging should be conducted 3 months after the operation to assess the bone‐tendon healing. The rehabilitation protocol should be adjusted based on bone‐tendon healing.

## DISCUSSION

As an arthroscopic stabilizing procedures for treating anterior shoulder instability, the advantage of the subscapular sling procedure is its relatively simple operation and fewer complications. In cases of mere anterior glenoid soft tissue damage, the glenoid labrum reconstruction (traditional Bankart repair) can be used, but it leads to a high risk of failure and further surgery.^9^, ^10^ For severe glenoid bone loss and residual dislocation after previous soft‐tissue stabilization, bone‐transferring procedures (Latarjet procedure) are performed, but it comes with potentially high complication rates, a steep learning curve, and risk of permanent nerve injury.^3^, ^9^ The subscapular sling procedure could fill the large gap between the glenoid labral reconstruction and bone‐transferring procedures. Biomechanical cadaveric and clinical pilot studies have been performed, and the subscapular sling procedure shows promising preliminary results.^6^, ^11^ It is believed that with the continuous improvement of technology, the surgical efficacy will continue to improve. Advantages and disadvantages are listed in Table 2. Pearls and pitfalls are listed in Table 3.

**TABLE 2:** Advantages and Disadvantages

Advantages	Disadvantages
The learning curve of arthroscopic technology is easier	Stabilize the shoulder solely through soft tissue repair surgery
Less invasive and with the least anatomical changes	Dependence on the complete integrity of the subscapular tendon
Portals lateral of conjoined tendon with less risk of nerve injury	The ankle function has been affected
Soft‐tissue reconstruction of the labrum with the graft	

**TABLE 3:** Pearls and Pitfalls

Pearls	Pitfalls
The tendon‐bone healing based on the bone tunnel is much more stable	Select the appropriate device and positioning to prevent the rupture of the bone tunnel
The half bundle of the peroneus longus tendon has sufficient strength and diameter	The half bundle of the peroneus longus tendon within the body must not be damaged
Only use 2 suture anchors and miniplate	More sutures can easily lead to confusion and interference
There are fewer incisions	There is a risk of neurovascular damage

The improvement of shoulder stability relies on the firm attachment of the graft.^12^ The common fixation method is to fix the graft on the bone surface with anchors. The residual soft tissue at the tendon‐bone interface, the tension of the graft, and local osteoporosis can all affect tendon‐bone healing. To achieve higher tendon‐bone healing strength, this technique inserts the graft ends into the glenoid bone tunnel. This technique can increase the contact area between the tendon and bone, and mesenchymal stem cells from the bone marrow can be better introduced to the tendon‐bone interface.^13^ The fixation of the graft through the bone tunnel is often used in ligament reconstruction, and clinical studies have confirmed its strong tendon‐bone healing ability.^14^ Previous experimental studies have also confirmed that repair based on the humeral tunnel is beneficial for tendon‐bone healing.^13^ With the development and improvement of arthroscopic instruments, the repair based on the humeral tunnel has been applied in clinical practice and holds great application potential.^15^

The selection of the graft is critical. The long head of the biceps tendon and the semitendinosus tendon are common sources of the graft.^12^, ^16^ As a pressure receptor of the shoulder, the long head of the biceps tendon can limit the displacement of the humeral head.^17^ To reduce the damage and influence on the shoulder, the long head of the biceps tendon should be retained. The semitendinosus tendon is not the best choice because of the limitation of the lateral decubitus position during graft preparation. The PLT has been widely used as a graft in previous studies and has the advantages of low cost, simple harvesting, and fewer complications compared with other grafts.^14^, ^18^, ^19^ In addition, the advantages of the PLT as a graft are its strength and diameter.^19^, ^20^ In this technique, the half bundle of the PLT already has sufficient strength and diameter as a graft, and the preserved half bundle of the PLT can reduce the impact on ankle function. Therefore, this study has recommended the use of a half bundle of the PLT as a graft.

In conclusion, this article describes an arthroscopic subscapular sling procedure based on the glenoid bone tunnel and half bundle of the PLT. The establishment of the glenoid bone tunnel can provide a more stable fixation for the graft, facilitating stable tendon‐bone healing. The half bundle of the PLT has sufficient strength and diameter and reduces the risk of complications.

## DISCLOSURES

The author (J.D.) declares the following financial interests/personal relationships which may be considered as potential competing interests: J.D. reports all support for the present manuscript: Supporting Program for Innovative Research Teams in Clinical Medicine during the 14th Five‐Year Plan 2022LCTD‐B25. The other authors (Y.G., J.G., X.B., M.Y., Y.Z., Z.Z.) declare that they have no known competing financial interests or personal relationships that could have appeared to influence the work reported in this article.

## FUNDING

This work was supported by National Natural Science Foundation of China (82172460).

## References

[1] Arner JW , Peebles LA , Bradley JP , Provencher MT . Anterior shoulder instability management: Indications, techniques, and outcomes. Arthroscopy. 2020;36:2791‐2793. 10.1016/j.arthro.2020.09.024 33172578

[2] Hurley ET , Aman ZS , Doyle TR , et al. Posterior shoulder instability, part II—Glenoid bone grafting, glenoid osteotomy, and rehabilitation/return to play—An international expert Delphi consensus statement. Arthroscopy. 2025;41:181‐195.e7. 10.1016/j.arthro.2024.04.034 38735411

[3] Hendy BA , Padegimas EM , Kane L , et al. Early postoperative complications after Latarjet procedure: A single‐institution experience over 10 years. J Shoulder Elbow Surg. 2021;30:e300‐e308. 10.1016/j.jse.2020.09.002 33010440

[4] Goodloe JB , Traven SA , Johnson CA , Woolf SK , Nutting JT , Slone HS . Increased risk of short‐term complications and venous thromboembolism in Latarjet‐Bristow procedures compared with Bankart repairs. Arthroscopy. 2021;37:806‐813. 10.1016/j.arthro.2020.10.039 33130058

[5] Hachem A‐I , Gonzalez‐Morgado D , Barraza G , et al. Arthroscopic iliac crest autograft glenoid augmentation using tape cerclage fixation for bony deficiency and recurrent anterior shoulder instability improves functional outcomes and achieves high union rates with graft resorption in nonloaded areas. Arthroscopy. 2025;41:4978‐4990. 10.1016/j.arthro.2025.04.042 40300734

[6] Vagstad T , Klungsøyr PJ , Drogset JO , et al. The novel arthroscopic subscapular sling procedure grants better stability than an arthroscopic Bankart repair in a cadaveric study. Knee Surg Sports Traumatol Arthrosc. 2020;28:2316‐2324. 10.1007/s00167-019-05737-3 31624904

[7] Klungsøyr JA , Vagstad T , Klungsøyr PJ , et al. The arthroscopic subscapular sling procedure results in low recurrent anterior shoulder instability at 24 months of follow‐up. Arthroscopy. 2024;40:2543‐2552.e1. 10.1016/j.arthro.2024.02.032 38453096

[8] Vagstad T , Klungsøyr JA , Bjerknes C , et al. Biomechanical comparison shows increased stability of an arthroscopic subscapular sling procedure compared to an open Latarjet reconstruction for anterior shoulder instability in specimens with major glenoid bone defect. J Exp Orthop. 2024;11:e70015. 10.1002/jeo2.70015 PMC11417344 39314811

[9] Shubert SB . *Editorial commentary*: Surgical treatment of shoulder instability with subcritical glenoid bone loss requires innovation: Bankart may risk significant recurrence and Latarjet may risk significant complications. Arthroscopy. 2021;37:2075‐2076. 10.1016/j.arthro.2021.03.055 34226000

[10] Delgado C , Calvo E , Valencia M , Martínez‐Catalán N , Luengo‐Alonso G , Calvo E . Arthroscopic Bankart repair versus arthroscopic Latarjet for anterior shoulder instability: A Matched‐pair long‐term follow‐up study. Orthop J Sports Med. 2025;13:23259671241313474. 10.1177/23259671241313474 PMC11909661 40092423

[11] Klungsoyr PJ , Guldal F , Vagstad T , Klungsoyr JA . A new subscapular sling operation to stabilize the shoulder. A cadaver study. J Exp Orthop. 2015;2:12. 10.1186/s40634-015-0028-y PMC4538717 26914880

[12] Klungsøyr JA , Vagstad T , Klungsøyr PJ , Hellevik AI , Drogset JO . Dynamic and static stabilization of anterior shoulder instability with the subscapular sling procedure. Arthrosc Tech. 2021;10:e1773‐e1781. 10.1016/j.eats.2021.03.027 PMC8322630 34336575

[13] Li X , Shen P , Su W , Zhao S , Zhao J . Into‐tunnel repair versus onto‐surface repair for rotator cuff tears in a rabbit model. Am J Sports Med. 2018;46:1711‐1719. 10.1177/0363546518764685 29620913

[14] Butt UM , Khan ZA , Amin A , Shah IA , Iqbal J , Khan Z . Peroneus longus tendon harvesting for anterior cruciate ligament reconstruction. JBJS Essent Surg Tech. 2022;12:e20.00053. 10.2106/JBJS.ST.20.00053 PMC9889288 36741045

[15] Tang J , Zhao J . Arthroscopic humeral bone tunnel‐based tendon grafting and trapezius transfer for irreparable posterior superior rotator cuff tear. Arthrosc Tech. 2021;10:e1079‐e1087. 10.1016/j.eats.2020.12.011 PMC8085408 33981554

[16] Kandeel AA . Soft arthroscopic latarjet procedure: Technical note on biceps tendon as a modified sling for restoration of glenohumeral stability. Arthrosc Tech. 2021;10:e1531‐e1537. 10.1016/j.eats.2021.02.021 PMC8252823 34258201

[17] Geng Y , Zhang Z , Tian Y , et al. Latissimus dorsi transfer combined with dynamic biceps rerouting for massive irreparable rotator cuff tear. Arthrosc Tech. 2025;14:103817. 10.1016/j.eats.2025.103817 PMC12598180 41220655

[18] Karapinar SE , Tas A , Dincer APR , Atay PT , Baydar PML , Kumbul H . An evaluation of the effect on ankle stability of the peroneus longus tendon graft used in anterior cruciate ligament reconstruction. Orthop J Sports Med. 2025;13:23259671251371229. 10.1177/23259671251371229 PMC12446790 40980563

[19] Opoku M , Abdramane AM , Abdirahman A , Fang M , Li Y , Xiao W . Can peroneus longus tendon autograft become an alternative to hamstring tendon autograft for anterior cruciate ligament reconstruction: A systematic review and meta‐analysis of comparative studies. J Orthop Surg Res. 2025;20:719. 10.1186/s13018-025-06080-9 PMC12308993 40734172

[20] Kumar K , Rao V , Panda AK , Joshi D . Peroneus longus tendon versus hamstring tendon autograft for primary anterior cruciate ligament reconstruction: A systematic review and meta‐analysis of comparative studies. Orthop J Sports Med. 2025;13:23259671251374313. 10.1177/23259671251374313 PMC12444037 40980562

